# Transcriptional Analysis of *Arabidopsis thaliana* Response to Lima Bean Volatiles

**DOI:** 10.1371/journal.pone.0035867

**Published:** 2012-04-25

**Authors:** Sufang Zhang, Jianing Wei, Le Kang

**Affiliations:** 1 State Key Laboratory of Integrated Management of Pest Insects and Rodents, Institute of Zoology, Chinese Academy of Sciences, Beijing, China; 2 Key Laboratory of Forest Protection, Research Institute of Forest Ecology, Environment and Protection, Chinese Academy of Forestry, State Forestry Administration, Beijing, China; Centro de Investigación y de Estudios Avanzados, Mexico

## Abstract

**Background:**

Exposure of plants to herbivore-induced plant volatiles (HIPVs) alters their resistance to herbivores. However, the whole-genome transcriptional responses of treated plants remain unknown, and the signal pathways that produce HIPVs are also unclear.

**Methodology/Principal Findings:**

Time course patterns of the gene expression of *Arabidopsis thaliana* exposed to Lima bean volatiles were examined using Affymetrix ATH1 genome arrays. Results showed that *A. thaliana* received and responded to leafminer-induced volatiles from Lima beans through up-regulation of genes related to the ethylene (ET) and jasmonic acid pathways. Time course analysis revealed strong and partly qualitative differences in the responses between exposure at 24 and that at 48 h. Further experiments using either *A. thaliana* ET mutant *ein2-1* or *A. thaliana* jasmonic acid mutant *coi1-2* indicated that both pathways are involved in the volatile response process but that the ET pathway is indispensable for detecting volatiles. Moreover, transcriptional comparisons showed that plant responses to larval feeding do not merely magnify the volatile response process. Finally, (*Z*)-3-hexen-ol, ocimene, (3*E*)-4,8-dimethyl-1,3,7-nonatriene, and (3*E*,7*E*)-4,8,12-trimethyl-1,3,7,11-tridecatetraene triggered responses in *A. thaliana* similar to those induced by the entire suite of Lima bean volatiles after 24 and 48 h.

**Conclusions/Significance:**

This study shows that the transcriptional responses of plants to HIPVs become stronger as treatment time increases and that ET signals are critical during this process.

## Introduction

A considerable amount of the carbon assimilated by plants is released back to the atmosphere as volatile organic compounds (VOCs), which often become even stronger after plants are attacked by herbivores. These VOC emissions after herbivore attack are often called herbivore-induced plant volatiles (HIPVs). Plant volatiles can mediate many important ecological processes [1], such as pollination, and indirect defenses in which natural enemies of the herbivores are attracted [2]. HIPVs also mediate plant–plant communication in the sense that plants attacked by herbivores can warn their intact neighbors of danger by emitting HIPVs [3].

Since the first reports on plant–plant communication in 1983 [3], [4], this phenomenon has been questioned [5], thoroughly investigated [6], [7], [8], [9], and experimentally proven [10], [11]. The molecular mechanisms and ecological relevance of plant–plant communication have attracted much interest from the research community, especially during the last 10 years [10], as a result of which its mechanisms have gradually emerged. Plant–plant communication is a common phenomenon in nature. Research has shown that volatiles can trigger the resistance of con-specific neighbors for almost 20 kinds of plants, including model species and economic crops [12], [13], [14], both in the laboratory [12], [15], [16] and under natural conditions [4], [6], [17], [18]. However, demonstrations of communication between interspecies are rare, with only three models having been reported to date [7], [17], [18], [19].

Although plant–plant communication has been proven in many systems, its molecular mechanisms, especially those of volatile perception and whole-genome transcriptions of receivers treated with volatiles from emitters [10], remain poorly understood. Although the location of HIPV receptors has not been fully identified yet, some studies have suggested that the jasmonic acid (JA) and ethylene (ET) pathways are involved [13], [20]. However, whether these two pathways are equally effective in the induction process is unknown. Knocking out certain biochemical pathways in receiver plants appears to be a helpful approach [21]. The gene transcript responses of receiver plants exposed to volatiles from emitters have been tested using custom microarray approaches covering part of the whole genome or that of hundreds of genes related to plant defense [12], [22], but the plant defense response is a systematic process involving numerous pathways and genes. Therefore, a time course study using a genome-wide microarray may provide more accurate information about the volatile response process.

Previous research has attempted to determine the extent to which volatile response and direct defense share similar gene expression profiles or pathways. By investigating the expression patterns of some defense genes, Kessler et al. [22] found that both processes activated the same set of genes but that direct damage induced much stronger responses in these genes. However, whether this correlation holds at the whole-genome level and exists in other model systems is yet to be seen. Other studies have also attempted to identify effective volatile chemicals from emitter plants. Although a significant body of evidence indicates that HIPVs, as a mixture, are an effective signal, whether individual compounds, mainly including green leaf volatiles and terenes, can also serve the same function is unclear. Some green leaf volatiles have been found to induce defense responses in several plants [9], [20], [23], [24], but whether these compounds can also act as an inducer in other systems has yet to be investigated.

In this study, a system including two model species was developed to investigate the communication dynamics between different plant species. Lima bean plants, a model species in plant–plant communication studies [10], [25], [26] and from which HIPVs can be effectively induced by leafminer feeding [27], were chosen as emitters. *Arabidopsis thaliana* plants, representing a well-established model with many mutants, were selected as receivers. Affymetrix ATH1 genome arrays were used to examine the gene expression patterns of HIPV-exposed *A. thaliana*, with the results showing that the responses of the receivers were positively correlated with treatment duration. Using *A. thaliana* mutants, we subsequently found that the ET pathway in the receiver plants is indispensable to communication. Furthermore, the volatile treatment-activated functional pathways were compared with those activated by direct feeding. Finally, we found that only several C_6_ compounds and terpenes of Lima bean volatiles can elicit similar genomic changes in *A. thaliana*.

## Results

### Communication between Lima bean and *A. thaliana*

**Figure 1:**
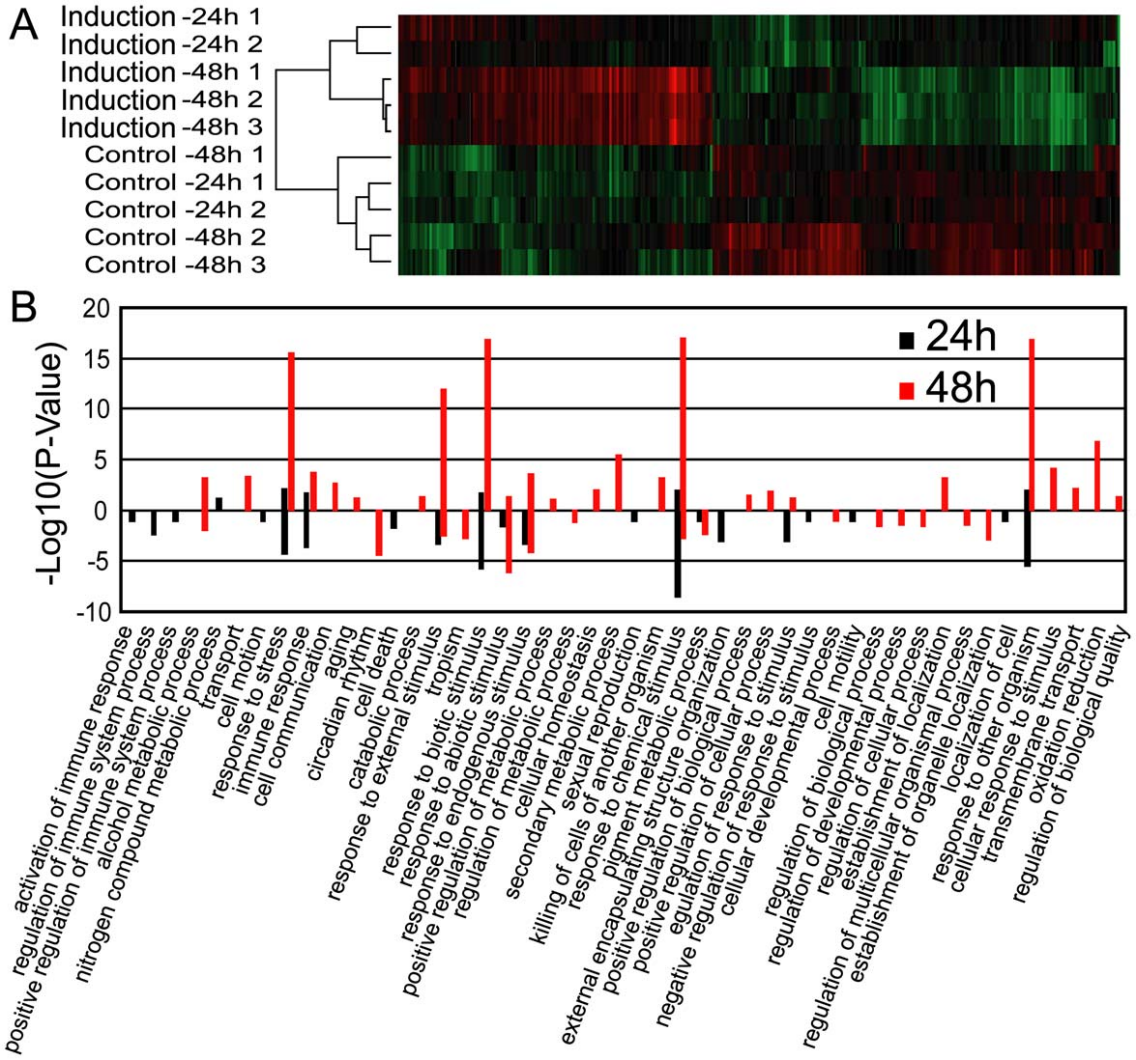
Transcriptional responses of *A. thaliana* to Lima bean volatiles after 24 h and 48 h of treatment. (A) Cluster analysis of the regulated genes in *A. thaliana* primed by volatiles from second instar larvae-damaged Lima beans. (B) GO enrichment analysis showing the differential expression of genes in *A. thaliana* (*p*<0.01). Values higher than zero on the *y*-axis refer to up-regulated genes, whereas those lower than zero refer to down-regulated ones. The GO terms were chosen at the third level.

To confirm whether communication occurs between Lima bean and *A. thaliana*, we examined the gene expression profiles of the receiver plants (*A. thaliana*) using the full-genome Affymetrix ATH1 microarray. qPCR tests were used to validate the reliability of this microarray (Figure S1). Treatment with volatiles from leafminer-damaged Lima bean plants lasted for 24 or 48 h; the 4-week-old unattached *A. thaliana* receivers were thus treated with the volatiles at two time intervals, whereas the control plants were treated with volatiles from healthy Lima bean plants. Results confirmed that, at the transcriptional level, *A. thaliana* responded to the volatiles from Lima bean plants infested with second instar larvae of leafminers; in addition, more genes were mobilized in the 48 h treatment than in the 24 h treatment (Figure 1A). Cluster analysis clearly showed that the gene expression patterns differed among the control, 24 h treatment, and 48 h treatment plants, suggesting that different sets of genes were affected by the treatments.

Enrichment analysis of Gene Ontology (GO) terms indicated that only a few gene groups were enriched in the 24 h treatment (Figure 1B). These genes were related to defense responses, such as response to stress, immune reaction, as well as biotic and chemical stimuli. However, more genes related to metabolic processes and other biological regulation processes were down-regulated. In contrast, after 48 h of treatment, a significant number of genes associated with both defense and metabolism pathways were up-regulated with greater amplification compared with the 24 h treatment. The defense pathways included genes responding to multiple organisms, stress, as well as external and chemical stimuli. The metabolism pathways involved genes related to alcohol metabolic processes, transport, cell communication, aging, catabolic processes, and secondary metabolic processes. Fewer genes were down-regulated after 48 h of treatment compared with the up-regulated ones, and these genes were associated with stimuli and biological regulation processes.

**Figure 2:**
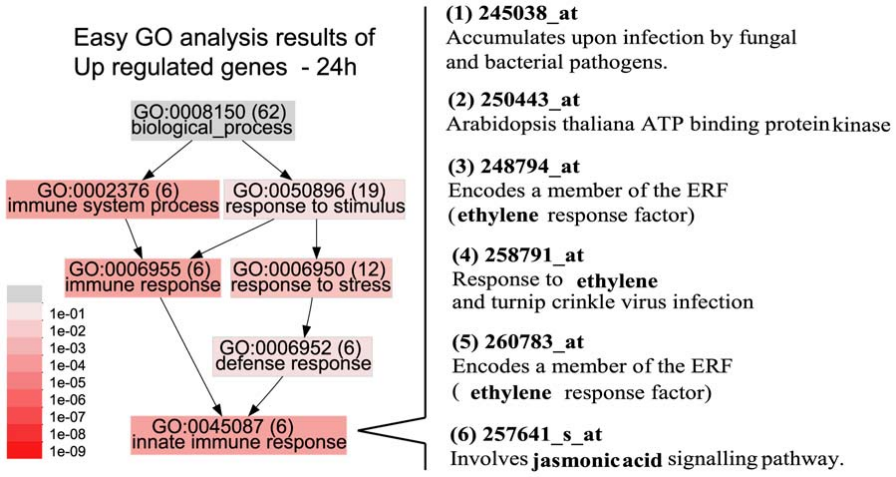
EasyGO analysis of the genes up-regulated in *A. thaliana* after 24 h of volatile treatment. The right panel lists gene information for the six innate immune response genes evaluated. Red, response to ET; blue, response to JA.

We focused more on the genes up-regulated by volatiles at the 24 h time point. Although only a few genes were up-regulated after 24 h of treatment, they were mostly related to defense responses, and these genes were continuously up-regulated after 48 h. Thus, the up-regulated genes may be related to the signal transduction that senses volatiles from neighbors. Analysis of the up-regulated genes by EasyGO (see Materials and Methods) showed that the genes related to the immune response pathway were up-regulated (Figure 2, left panel). Of the six up-regulated immune response genes, three were related to ET response and one was related to JA response (Figure 2l, right pane).

### ET and JA pathways underlie the detection of HIPVs

**Figure 3:**
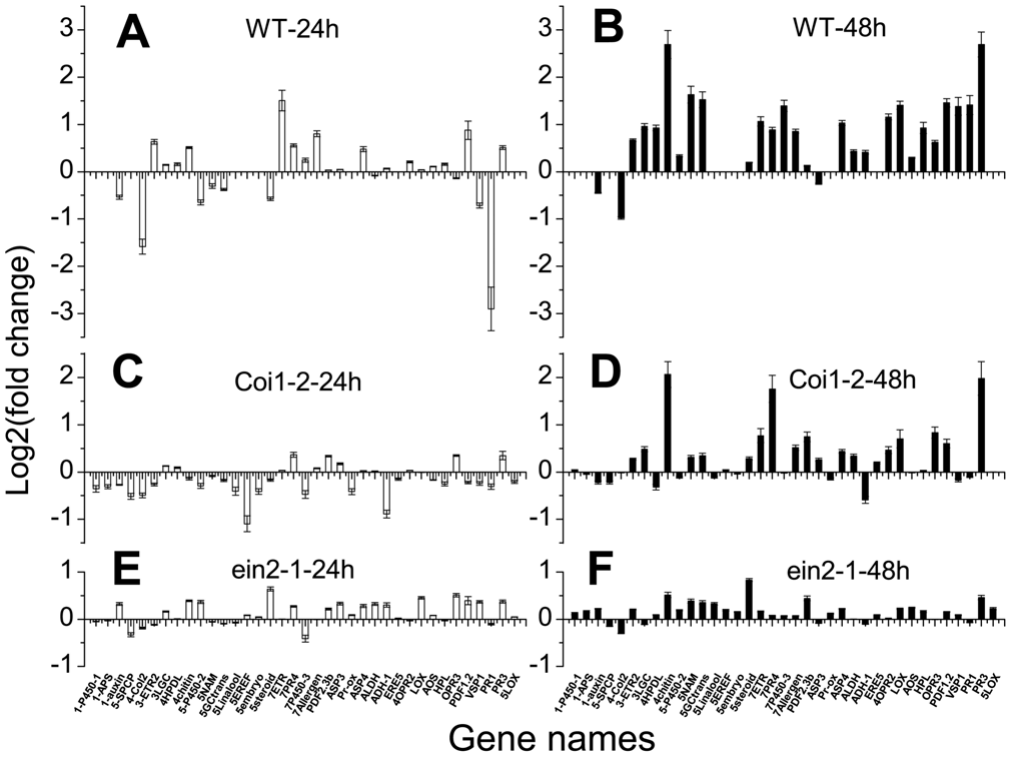
Responses of wild-type as well as *coi1-2* and *ein2-1* mutant *A. thaliana* to the volatiles. Expression patterns of 37 selected genes in the wild-type as well as *coi1-2* and *ein2-1* mutant *A. thaliana* plants after 24 or 48 h of treatment with volatiles from leafminer-infested Lima beans are shown. (A) Response of wild-type *A. thaliana* after 24 h of treatment with volatiles from leafminer-infested Lima beans. (B) Response of wild-type *A. thaliana* after 48 h of treatment with volatiles from leafminer-infested Lima beans. (C) Response of *coi1-2 A. thaliana* after 24 h of treatment with volatiles from leafminer-infested Lima beans. (D) Response of *coi1-2 A. thaliana* after 48 h of treatment with volatiles from leafminer-infested Lima beans. (E) Response of *ein2-1 A. thaliana* after 24 h of treatment with volatiles from leafminer-infested Lima beans. (F) Response of *ein2-1 A. thaliana* after 48 h of treatment with volatiles from leafminer-infested Lima beans.

Based on the results shown in Figure 2, we hypothesized that the ET and JA pathways were involved in the communication between Lima bean and *A. thaliana*. To confirm this, we used two *A. thaliana* mutants as receiver plants, namely, *coi1-2* (a JA response-deficient mutant) and *ein2-1* (a mutant insensitive to ET). According to the response magnitude of the microarray results, 37 genes were selected to examine the induction effects of Lima bean volatiles on these mutants (Supporting Methods S1; Table S2). Table S1 shows the primers (full and short names). After 24 and 28 h of HIPV treatment, the *coi1-2* mutant could still respond to the HIPVs, although the magnitude of change was much lower than that observed in the wild-type plants (Figure 3A–D). Furthermore, *ein2-1* was nearly “deaf” to HIPVs, and the genes showed much lower responses than the *coi1-2* mutant even after 48 h (Figure 3E and F). These results suggest that both ET and JA pathways are involved in detecting HIPVs and that ET is indispensable to the communication between Lima bean and *A. thaliana*.

### Gene expression profiles of *A. thaliana* in response to leafminer feeding and induction volatiles

Although the gene expression patterns of *A. thaliana* in response to Lima bean HIPV treatment have been characterized, little is known about how these patterns are related to those caused by direct insect feeding. Response to larval feeding in *A. thaliana* was tested using the same GeneChip as for the volatile exposure experiment. Unsurprisingly, the gene expression profiles of leafminer-infected tissue significantly differed from those of the control tissue. Approximately 3096 genes were regulated, of which 1695 were up-regulated and 1401 were down-regulated, with a threshold of more than twofold change. GO enrichment analysis indicated that feeding induced significant modifications in the genes mainly associated with stimulus-response and metabolic processes (Figure S2). Up-regulated genes were found in many processes related to responses to biological and organic chemical stimuli, and processes involved in immune and defense responses. The metabolic up-regulation by leafminer feeding was related to amending mechanisms, such as processes involved in cell wall macromolecule metabolism and catabolism. The down-regulated genes, on the other hand, were related to pigment biosynthetic processes, carbohydrate metabolic processes, and response to radiation. These results suggest that upon direct damage plants may devote more resources to deal with tissue wounds and reduce their energy consumption in basic metabolic and stimulus-response processes not related to defense.

**Figure 4:**
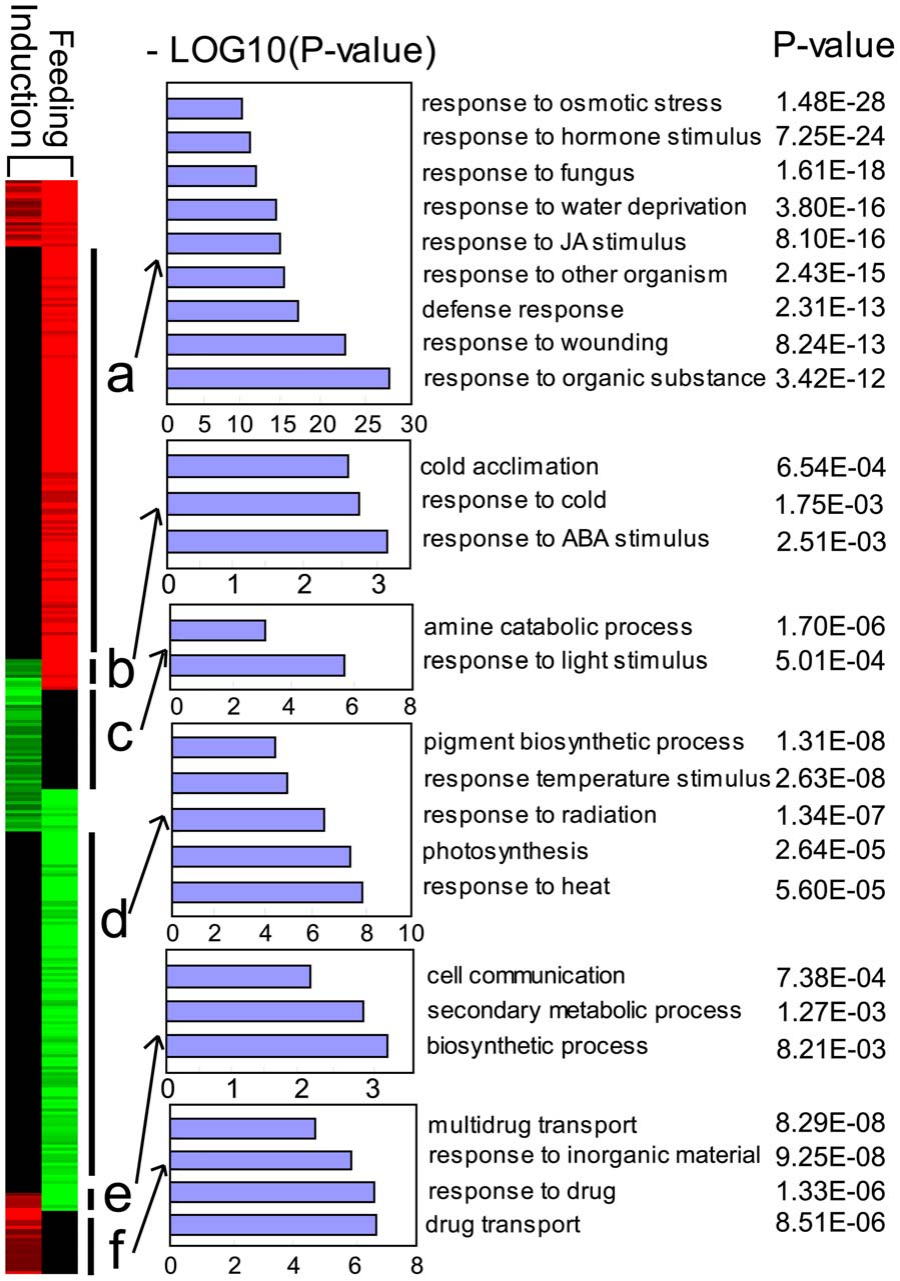
Comparison of the genomic responses in *A. thaliana* after treatment with HIPVs and leafminer feeding.

To further characterize the genes related to the exposure process, we compared the gene expression profiles obtained from the 48 h treatment with Lima bean volatiles and insect feeding experiments (Figure 4). Cluster analysis was achieved using the identified “differentially expressed genes” of the volatile exposure experiment and leafminer infestation treatment (Figure 4). Moreover, cluster analysis of gene expression profiles showed a clear separation between the patterns caused by direct feeding and those caused by volatile induction, suggesting that the transcriptional response to feeding is not a simple augmentation of the activities of the genes that were primed by volatiles. Instead, new sets of genes were activated as a result of feeding damage. A significant number of genes involved in stress response and resistance to harsh environmental conditions were up-regulated by leafminer feeding but not triggered by volatile induction, suggesting that leafminer feeding is a stress factor and can activate the defense pathways (Figure 4, cluster A). On the other hand, some genes involved in the secondary metabolism and cell communication were up-regulated by volatile induction but down-regulated by feeding, whereas some other genes involved in response to abscisic acid and cold environment were up-regulated by feeding but down-regulated by volatile induction (Figure 4, clusters B and E). Furthermore, feeding down-regulated the expression of some genes related to such environmental factors as temperature, radiation, and photosynthesis; however, these genes were not affected by the induction process (Figure 4, cluster D). On the contrary, some genes showed changes specific to volatile induction (not affected by feeding), which included pathways related to processing inorganic substance and drug transport (Figure 4, cluster F). Finally, we also found that both feeding and volatile induction processes up-regulated a set of genes participating in response to biotic factors (fungi and other organisms) and to wounding, defense, and the amine metabolic process. Conversely, feeding and volatile induction both down-regulated plant response to auxin stimulus, which is an important regulatory hormone of plant growth [28].

### Effects of individual compounds and Lima bean volatiles on *A. thaliana*

**Figure 5:**
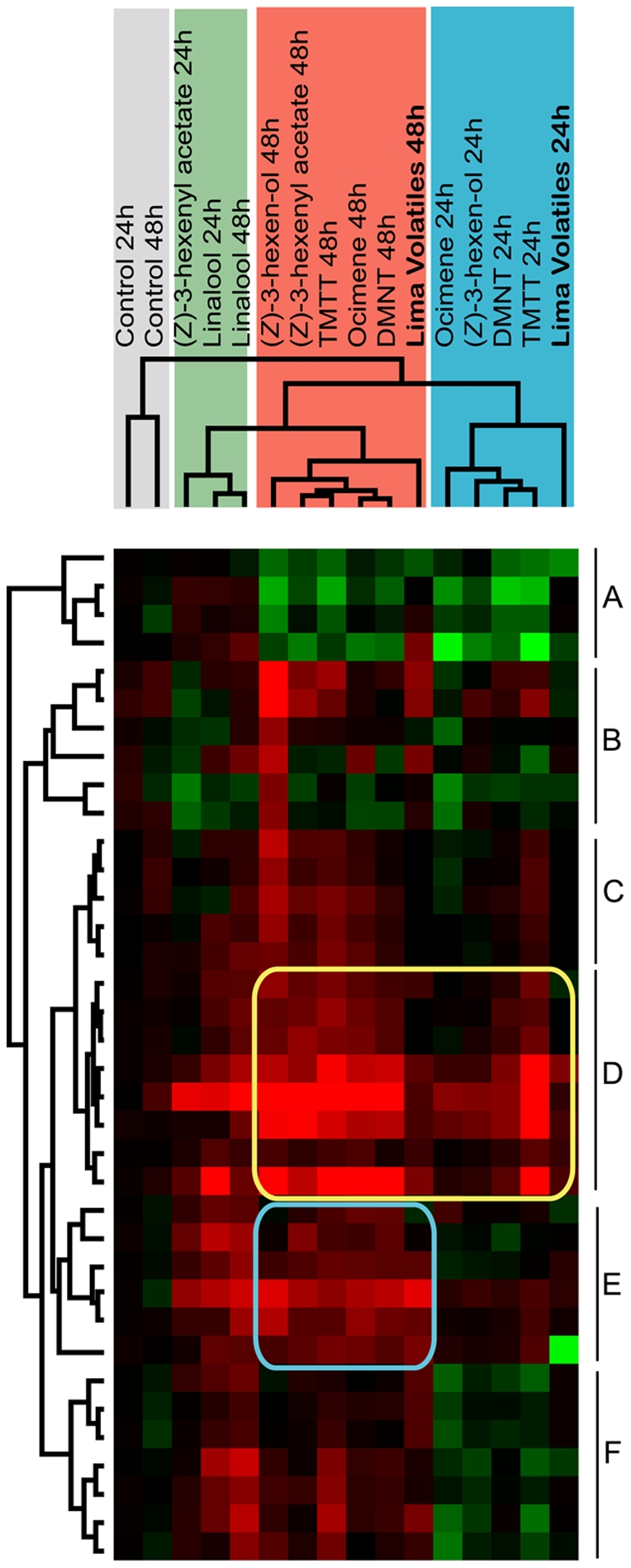
Comparison of primer effects between synthetic chemicals and Lima bean volatiles. Hierarchical clustering of the expression patterns of selected genes in *A. thaliana* treated with synthetic chemicals and Lima bean volatiles (24 or 48 h). The blue area represents the cluster of genes similarly affected by the 24 h treatment with Lima bean volatiles; pink area, cluster of genes similarly affected by the 48 h treatment; green, cluster of Lima bean volatiles; gray, cluster of healthy control plants. The clustering results along the *y*-axis of the color matrix reveal six categories of genes (A–F) showing similar reactions among the variety of treatments.

To analyze the induction effects of individual compounds, we selected two green leaf volatiles [(*Z*)-3-hexen-ol and (*Z*)-3-hexenyl acetate] and four terpenes [linalool, ocimene, (3*E*)-4,8-dimethyl-1,3,7-nonatriene (DMNT), and (3*E*,7*E*)-4,8,12-trimethyl-1,3,7,11-tridecatetraene (TMTT)], which comprise nearly 95% of all VOCs detected in the headspace of leafminer-damaged Lima beans (Figure S3; Supporting Methods S1). The genes tested here — a total of 37 genes that reacted to the 24 and/or 48 h volatile induction treatments — were the same as those evaluated in part 2 (Table S1). In comparing the responses of *A. thaliana* induced by the testing compounds with those induced by the volatiles from Lima beans infested with leafminers (see Materials and Methods), we found that ocimene, DMNT, TMTT, and (*Z*)-3-hexen-ol were similarly effective as the Lima bean volatiles both in the 24 h treatment (Figure 5, blue area) and in the 48 h treatment (pink area). Linalool and (*Z*)-3-hexenyl acetate, on the other hand, were clustered into a different group. Four genes related to metabolism were down-regulated after being treated with ocimene, DMNT, TMTT, (*Z*)-3-hexen-ol, and the Lima bean volatiles for 24 or 48 h, but only slightly regulated by Linalool and (*Z*)-3-hexenyl acetate (Figure 5, gene cluster A), suggesting that Linalool and (*Z*)-3-hexenyl acetate have minimal influence on plant metabolism and development.

Genomic responses induced by ocimene, DMNT, TMTT, and (*Z*)-3-hexen-ol also showed time dependence. Some genes (Figure 5, cluster D) were markedly up-regulated after 24 or 48 h of treatment, and half of them were ET response factors. These findings suggest that the ET signal pathway may play a crucial role in the early recognition and continuous processing of induction volatiles. Several JA pathway-related genes (Figure 5, gene cluster F) were down-regulated after 24 h but up-regulated after 48 h, suggesting that the JA pathway may participate in processing volatiles after the recognition phase. Furthermore, some defense-related genes (Figure 5, clusters C and E) were slightly regulated after 24 h but significantly up-regulated after 48 h of treatment, indicating a time-dependent defense response in these plants. Several stress response and transcript regulation genes (Figure 5, cluster B) did not exhibit a clear expression pattern over time.

## Discussion

The arms race between plants and herbivorous insects has driven diverse defense strategies in plants over time. The ability of receiver plants to detect volatiles released from neighboring plants under herbivore attack and subsequently adjust their alert levels (i.e., plant–plant communication) seems to be an adaptive mechanism [11]. Our results showed that communication can indeed take place between plants of different species, consistent with previous reports [22].

We used Affymetrix microarrays (*Arabidopsis* ATH1 genome array) containing nearly all the genes to test the effects of plant–plant communication on receivers. Although much research has shown that VOCs can induce the expression of some crucial defense genes, the system response of receivers remains unclear [12]. Some studies have used microarrays [22], but the detection of the number of genes was restrained in these researches. Thus, use of a full-genome microarray is essential for monitoring a large fraction of the transcriptome and grasps the real responses of plants [29]. Our research holistically depicts the effects of communication on receivers. With 24 h of communication, the immune responses mainly related to ET and JA were regulated, but the SA-related signal was restricted, which are in congruence with the results of previous studies showing that SA and JA signals are in conflict under many conditions [30]. When the treatment time was increased to 48 h, the defense genes were significantly regulated, containing nearly all stimulus-response conditions and some secondary materials. However, the response to auxin was slightly restricted (Figure S4), which many suggest to be a tradeoff between defense and growth [31].

As the ET and JA signal pathways serve as initial communication response signals, we used mutants of these two signals to detect the possible receptor signals of the chemicals from neighbors. Results indicated that the ET response signal is crucial to chemical response in plant–plant communication. The JA mutant has a weaker response to chemicals. Accordingly, Ruther and Kleier [20] demonstrated that ET can synergize volatile emission in maize induced by exposure to (*Z*)-3-hexen-1-ol. The consanguineous relationship between ET and JA signals has been thoroughly characterized [32], [33]: they can either function together or act independently, and different mutants in the pathways have different phenotypes [34]. The ET response signal is perhaps a receptor of the chemicals from the neighbors, with the response subsequently mobilizing ET and JA downstream defense genes through the interaction of the two plant hormones.

The receiver's speed of response to volatiles from its neighbors may be crucial for plants to cope with herbivore attack. Our results showed that the immune responses related to the ET pathways can be up-regulated after 24 h of exposure and that defense genes, including those responsible for nearly all stimulus-induced responses and for the production of some secondary substances also important for plant defense, were significantly up-regulated after 48 h of exposure (Figure 1B). Although plants can respond to direct damage within a couple of hours [35], they seem to take 2–3 days to initiate response and fully develop their cellular defense mechanisms against volatiles. The necessity of such dual-reaction systems is likely related to a growth-defense tradeoff [36], [37].

The speed–accuracy tradeoff is another dimension of the volatile response process. A complex blend of HIPVs may accurately encode the damage incurred by a plant, but it may require more resources and a longer time for a receiver plant to decode. Therefore, the identification of active single volatiles is essential. Most studies that identified volatiles acting in plant–plant communication focused on green leaf volatiles or termenes separately [14], [23]. The present study carried out a relatively comprehensive analysis of each member from one kind of emitter. Active volatiles are apparently correlated with their emission rhythms. Our results showed that some single compounds can be functionally similar to the entire set of Lima bean volatiles in *A. thaliana* induction. An ability to respond to emitters' signals all day long would be beneficial to plants. We have previously found in our study on the Lima bean–leafminer system that (*Z*)-3-hexen-ol is mainly released at night, whereas terpenes are abundantly released during the day [38]. Therefore, a plant that can be primed by both molecules, such as *A. thaliana*, would have better chances of rapidly preparing itself for a real attack because it may pick up signals anytime. However, the effective volatiles may vary for different plants, as (*Z*)-3-hexenyl acetate can elicit defense responses in maize, Lima bean, and hybrid poplar [9], [24], [25]. Furthermore, the effects of linalool differ between receiver plants, with the likely reason being that linalool is commonly released by volatiles from flowers, thereby restricting its accuracy as a signal of insect damage [39], [40].

In comparing genomic responses to volatiles and those to feeding damage, we found that the feeding-induced response in *A. thaliana* was not a simple augmentation of the gene activation caused by volatile exposure and that new sets of genes were recruited (Figure 4). Direct feeding and volatile treatment both regulated a considerable number of biotic and abiotic defense genes, but direct feeding activated more genes and induced greater gene expression fold change. These indicate that a greater extent of plant defense is initialized after herbivore feeding. Feeding damage down-regulated many basic metabolic genes and up-regulated defense genes, suggesting that there may be a tradeoff between growth and defense, such that plants devote more resources to defense when severe damage occurs.

In summary, our data show that HIPVs can prime receiver plants, a process that is distinct from the defense response caused by direct feeding. The induction process starts with up-regulation of immune pathways (after 24 h of treatment), followed by significant enhancement of cellular pathways underlying responses to biotic and abiotic stimuli (after 48 h of treatment). Only some single compounds are as effective as the whole set of Lima bean volatiles in inducing *A. thaliana*. Feeding damage, on the other hand, activates new sets of genes with greater fold changes. The ET and JA pathways are involved in the induction process in different degrees, with the ET pathway specifically necessary for sensing the induction signal in the early phase of induction.

Plant–plant communication is being increasingly appreciated [41]. Our data provide a genomic basis for this phenomenon, and this study has identified many biochemical pathways linked to gene expression patterns. However, further investigation is needed to clarify the physiological function of induction responses in receivers upon herbivore attack.

## Materials and Methods

### Plants and insects

Lima bean (*Phaseolus lunatus* L. cv Sieva) was individually seeded in plastic pots (12 cm diameter) with peat/vermiculite (3∶1) medium in an environmental chamber. Bean plants with two fully developed primary leaves were used in all experiments.

The wild-type and mutant *A. thaliana* plants used in this study were in the Col-0 background. The *coi1-2* and *ein2-1* mutants have been previously described by Xu et al. [42] and Roman et al. [43], respectively. The seeds were surface sterilized for 15 min in 10% bleach, washed five times with sterile water, and plated on half-strength Murashige–Skoog medium [44]. Plants were stratified at 4°C for 2 days in the dark and then transferred to an environmental chamber set at 22°C with a 16 h light/8 h dark cycle (light intensity, 120 µmol m^−2^ s^−1^). After 2 to 3 weeks, seedlings were also potted in peat/vermiculite (1∶2) medium and then placed in a growth room at 22°C with a 16 h light/8 h dark cycle (light intensity, 120 µmol m^−2^ s^−1^). Four-week-old *A. thaliana* plants were specifically used in our experiments. Pea leafminer (*Liriomyza huidobrensis*) was cultured under laboratory conditions for 3 years. Lima bean was used as the host plant of *L. huidobrensis* for rearing.

### Induction effect of leafminer-induced Lima bean volatiles

Two-week-old Lima bean plants containing more than 50 second instar leafminer larvae were used as emitters, whereas 4-week-old unattached *A. thaliana* plants served as receivers. For the volatile transportation system, compressed air (Beijing Gas Main Plant, Beijing, China) was pushed through three glass bottles (500 ml) containing molecular sieve (0.5 nm; Beijing Chemical Company, Beijing, China), freshly activated charcoal (Beijing Chemical Company), and distilled water, respectively. The filtered and moisturized air was pushed into a glass jar with a shape similar to that of a desiccator (15 l). The air was absorbed with Porapak Q (80–100 mesh size; Supelco, USA) and tested with an Agilent gas chromatographer (GC) (6890N) coupled with a mass spectrometry (MS) system (5973 MSD; Agilent Technologies, Inc., USA); air clarity was ensured to avoid the interference of pollution [45]. The emitter plants (more than three Lima beans in each pot) were placed at the bottom of the jar, whereas the receiver plants were placed on a stainless steel shelf, such that the receivers were above the emitters and immersed in the headspace of the Lima bean plants. At the end of the system was a membrane pump (Beijing Institute of Labour Instruments, China). The compressed air was released at low speed (50 ml/min) by the pump. The low air speed ensured that the volatiles from the emitters would remain in the jar at a relatively high concentration for a long duration. By using this positive/negative pressure system, we ensured that no ambient air was sucked into the jar, although the plants could get fresh air, thus avoiding the normal restrictions of a closed system [21]. The control receiver samples were treated with healthy Lima bean plants. After 24 or 48 h, the receivers were frozen in liquid nitrogen. Each repeat contained tissue from at least eight plants, and more than two repeats were prepared.

### Leafminer feeding experiment

More than 200 mated *L. huidobrensis* adults were released onto 4-week-old *A. thaliana* leaves for oviposition and then removed within 4 h. After the leafminers grew to the second instar (96 h after oviposition), leaves damaged by leafminer larvae were selected as the leafminer-infected samples (at least 50 larvae were found in the Lima bean leaves). Three separate samples containing tissue from at least eight plants were prepared from the leafminer-infected leaves. Three control samples were also prepared from the leaves of healthy plants of the same age.

### Induction effect of synthetic chemicals

*Arabidopsis thaliana* plants were placed in the same jar (15 l) used in the plant–plant communication setup, and 5 µl of 0.5 mM synthetic chemicals in dichloromethane was injected onto a cotton ball (4 mm diameter). The cotton ball was bound to the steel shelf in the jar. The same amount of dichloromethane was used as control. Finally, the *A. thaliana* plants were frozen in liquid nitrogen. Each sample contained tissue from at least eight plants, with four replicates.

Two green leaf volatiles [(*Z*)-3-hexen-ol and (*Z*)-3-hexenyl acetate] and four terpenes (linalool, ocimene, DMNT, and TMTT), which comprise nearly 95% of all VOCs detected in the headspace of leafminer-damaged Lima beans, were selected to analyze the induction effects of individual compounds. Most of the compounds used in this experiment were purchased commercially: (*Z*)-3-hexen-ol (≥95% pure) and linalool (97% pure) were obtained from Sigma-Aldrich (St. Louis, MO); (*Z*)-3-hexenyl acetate (99% pure) was obtained from Tokyo Kasei Kogyo Co. (Tokyo, Japan); and ocimene (≥75% pure) was obtained from Fluka (Buchs, Switzerland). DMNT and TMTT were kindly provided by Dr. W. Boland of the Max Planck Institute for Chemical Ecology (Jena, Germany).

### Preparation of cDNA sample

Total RNAs were isolated using an RNeasy® Plant Mini Kit (Qiagen, Valencia, CA, USA). Each sample for RNA extraction contained tissue from at least eight *Arabidopsis* plants. cDNA was prepared following the manufacturer's instructions (www.affymetrix.com/support/technical/manual/expression_manual.affx).

### Microarray hybridization and data analysis

Affymetrix microarrays (*Arabidopsis* ATH1 genome array) containing 22,810 probe sets were used in our experiments. Labeling and hybridization on the ATH1 microarrays (one sample per chip) were performed according to the manufacturer's instructions (www.affymetrix.com/support/technical/manual/expression_manual.affx). Global analysis of temporal gene expression was performed by subjecting the absolute expression values for scaling using Affymetrix MAS5.0. The probe arrays were further analyzed with GENESPRING Version 5.0 (Silicon Genetics). Normalization of every gene and chip was performed to allow comparisons of two or three independent replicates performed for each set of experiment. SAM analysis (Significance Analysis of Microarrays software package) was conducted for *A. thaliana* triplicate samples between treatment and control plants using a *q* value ≤0.05 and a fold change cutoff ≥2 to identify the genes differentially expressed in the treatments [46]. We searched GO enrichment information for the differently expressed probe sets using EasyGO (http://bioinformatics.cau.edu.cn/easygo/category_treeBrowse.html). We applied *χ* ^2^ analysis for the biological process search, and the cutoff for false discovery rate (FDR) was adjusted using a *p* value of 0.0001. Cluster 3.0 and TreeView were used (http://rana.Stanford.EDU/software/) to group and display genes with similar expression profiles [47]. We used the default options of hierarchical clustering with uncentered correlation similarity metrics. All GeneChip data sets are available in a MIAME-compliant format through GEO (accession no. GSE33505).

### Real-time PCR

PCR was performed in 20 µl of reaction volume containing 10 µl of 2× SYBR® Premix EX Taq™ Master Mix (TaKaRa, Kyoto, Japan), 5 µM concentration each of gene-specific primers (Table S1), and 1 µl cDNA templates. Reactions were carried out on an Mx 3000P detection system (Stratagene, La Jolla, CA, USA). The following thermal cycler parameters were used to produce the melting curves, which were in turn used to assess the specificity of the PCR products: 2 min at 95°C; 40 cycles of 5 s at 95°C, 20 s at 58°C, and 20 s at 72°C; and 1 cycle of 30 s at 95°C, 30 s at 58°C, and 30 s at 95°C. β-Actin was used as the housekeeping gene. A standard curve was derived from the serial dilutions of plasmid containing the target DNA segment to quantify the copy numbers of target mRNAs. The amount of each gene was then normalized to the abundance of β-actin. Subsequently, the normalized values of each gene in the stressed samples were divided by those in the untreated controls, and the folds were used as the relative levels of each gene.

## Supporting Information

Figure S1 **Validation of microarray data by qPCR.** Scatter plot showing a positive correlation of gene expression patterns between qPCR and microarray hybridization. The *x*-axis represents microarray hybridization, whereas the *y*-axis represents qPCR.
(EPS)

Figure S2 **GO enrichment analysis of genes regulated by leafminer feeding in** ***A. thaliana*** **(** ***p*** **<0.01).** The GO terms were chosen at the third level.
(EPS)

Figure S3 **Absolute amount volatiles from emitters.** Absolute amount of detected volatiles emitted from second instar leafminer-infested Lima bean plants at different times of the day.
(EPS)

Figure S4 **GO enrichment of down-regulated genes in** ***A. thaliana*** **after 24 h of treatment.** The graph displays term enrichment levels along with the GO term hierarchy within the biological process branch, and the analysis was performed using EasyGO. The classification terms and their serial numbers are shown as rectangles. The numbers in brackets represent the total number of genes that may be involved in the corresponding biological processes. The graph displays the classification term enrichment status and term hierarchy. The color scale shows the *p* value cutoff levels for each biological process. The darker colors represent the more significant biological processes in the putative stigma pathway.
(EPS)

Table S1 **Primers used for qPCR.**
(XLS)

Table S2 **Five most significantly enriched GOs in** ***A. thaliana*** **up-regulated by 48 h of volatile treatment.**
(XLS)

Methods S1 **Selection principle of tested genes, and method for lima bean volatile collections, identification, and quantification.**
(DOC)
