# Supplementary material for: Transcriptional Analysis of Arabidopsis thaliana Response to Lima Bean Volatiles
Source: PLoS One. 2012 Apr 25;7(4):e35867. doi: 10.1371/journal.pone.0035867 (PMC3338473; doi:10.1371/journal.pone.0035867)
Supplement: Methods S1 — Selection principle of tested genes, and method for lima bean volatile collections, identification, and quantification. (DOC) [file pone.0035867.s007.doc]

**Supplement methods**

**1,** **Selection of genes tested by Q-PCR**

Based on the response magnitude of the microarray results, we selected some representative genes, which were used to detect the response of mutant plants and effect of single chemicals. These genes were key members involved in some important signal pathways of plant such as defense, immune reaction, metabolism and development, with β-actin as the house-keeping gene. Table S1 gives their primers, full names, and short names.

The genes were selected according to the following rules. Firstly, we selected five most significantly enriched GOs in *Arabidopsis thaliana* that were up regulated by 48h volatile treatment, which were “response to chemical stimulus”, “response to other organism”, “response to biotic stimulus”, “response to stress”, “response to external stimulus” (shown in table S2). The genes included in all the five GOs and with a fold change more than 2 were selected. Here, we got 7 genes, which were 256243_AT, 266385_AT, 249052_AT, 245928_S_AT, 265530_AT, 260783_AT, 252618_AT. Secondly, twelve genes that were most significantly up regulated by leafminer feeding in *Arabidopsis thaliana* were selected, but the ones that we don’t know their functions were removed. Here, we got more 7 genes, which were 252368_at, 249527_at, 250662_at, 260203_at, 245928_s_at, 264886_at, 264415_at. Thirdly, our analysis indicated that JA and ET pathways were important in the defense response of *Arabidopsis thaliana*, thus the key genes of these two pathways were included in our gene list. Here, we got more 7 genes, which were 257922_at, 261470_at, 248799_at, 259875_s_at, 249208_at, 245253_at, 261037_at. Fourthly, we selected 3 down regulated genes, which were 265665_at (the most significantly down regulated genes in *Arabidopsis thaliana* by leafminer feeding), 259790_s_at (auxin response gene, an important gene related to growth), 258497_at (putative flowering-time gene CONSTANS (COL2)). Fifthly, 6 gene that were medially regulated by volatile treatment or leafminer feeding in *Arabidopsis thaliana* were selected randomly, which were 253073_at, 266267_at, 258791_at, 256589_at, 245038_at, 264758_at. Lastly, 7 genes that were not significantly regulated by volatile treatment or leafminer feeding in *Arabidopsis thaliana* were selected randomly, which were 264372_at, 245253_at, 266118_at, 250385_at, 262646_at, 263157_at, 261712_at.

**2, Lima bean volatile collection**

Three collections of volatiles were used: (1) clean bags - the odor collected from a clean oven bag (Reynolds Oven Bags, Reynolds Kitchens, Richmond, VA, USA) was used as a control; (2) healthy Lima beans; and (3) damaged plants –second instar leafminer (96 h after oviposition) damaged Lima beans.

The headspace volatile collection setup was prepared similarly as that described previously (Wei *et al.* 2006). To measure the dynamic patterns of plant volatile emission, a whole day was divided into 3 time intervals: 08:00-15:00 (morning), 15:00-22:00 (afternoon), and 22:00-08:00 in the next day (night). The absorbing glass collectors connecting to each bag were replaced with a new one in each time interval. All aeration extracts were stored at -20°C until used in chemical analyses or behavioral experiments. The plants were weighed immediately after being collected. Numbers of leafminer larvae on the leaves were recorded.

**3, Chemical identification and quantification**

Collected volatiles were identified similarly as described previously (Wei *et al.* 2006). And a different GC (7890A; Agilent Technologies, Inc., Santa Clara, CA, USA) coupled with an auto-inlet installation was used to quantify the collected volatiles. The system was equipped with the same DB-WAX column and the same template program as the previous GC-MS system. Heptanoic acid, ethyl ester and dodecanoic acid, ethyl ester (1 ng μL^-1^, 5 ng μL^-1^, 20 ng μL^-1^, 50 ng μL^-1^ and 100 ng μL^-1^) were used as external standards for developing standard curves to quantify volatiles in the samples.

**References**

Wei J.N., Zhu J. & Kang L. (2006). Volatiles released from bean plants in response to agromyzid flies. *Planta*, 224, 279-87.
